# Supplementary material for: Family meals with young children: an online study of family mealtime characteristics, among Australian families with children aged six months to six years
Source: BMC Public Health. 2017 Jan 24;17:111. doi: 10.1186/s12889-016-3960-6 (PMC5259977; doi:10.1186/s12889-016-3960-6)
Supplement: Additional file 1: Table S1. — Family Meals with Young Kids survey questions, response scales and sources used to inform development of questions and response items. (DOCX 40 kb) [file 12889_2016_3960_MOESM1_ESM.docx]

**Title:** Family meals with young children: an online study of family mealtime characteristics, among Australian families with children aged six months to six years

**Authors:** Eloise-kate Litterbach**^1^**, Karen J Campbell PhD^1^, Alison C Spence PhD^1^

^1^Institute for Physical Activity and Nutrition, School of Exercise and Nutrition Sciences, Deakin University, 221 Burwood Hwy, Burwood, Victoria, Australia 3125

**Email addresses:**

Eloise-kate Litterbach: [e.litterbach@deakin.edu.au](mailto:e.litterbach@deakin.edu.au)

Karen J Campbell: [karen.campbell@deakin.edu.au](mailto:karen.campbell@deakin.edu.au)

Alison C Spence: [a.spence@deakin.edu.au](mailto:a.spence@deakin.edu.au)

**Corresponding Author:**

Eloise-kate Litterbach

Institute for Physical Activity and Nutrition, School of Exercise and Nutrition Sciences; Deakin University

221 Burwood Hwy, Burwood Victoria Australia 3125

Phone: 0425860714

Email: e.litterbach@deakin.edu.au

## Abstract

## Background: Evidence suggests that family meals influence food intakes and behaviours, which in turn impact children’s eating habits, diets and health. Mealtimes therefore offer potential as settings for health promotion. Given diet, health behaviours and health are often socioeconomically patterned, it is important to consider whether family meals differ by socioeconomic position (SEP).

**Methods:** The Family Meals with Young Kids study was an online survey completed by parents in 2014. Mealtime characteristics measured included; frequency of shared meals across the day, duration and location of mealtimes, parental modelling, and parental perceived importance of the evening meal. Maternal education was used to assess SEP. The aims of this study were to describe family meal characteristics among Australian families with children aged six months to six years and to describe the socioeconomic patterning of these.

**Results:** Participants (n=992) were mostly mothers (97%) with a university degree (71%). The evening meal was the most frequently reported meal eaten together with the responding parent and child (77% ≥five nights/week). Snacks were least commonly eaten together (39% ≥five days/week). The frequency of having everyone present for the evening meal was inversely associated with SEP (OR 0.70, CI 0.54-0.92). Parent rated importance of family meals was generally high and positively associated with higher SEP (OR 1.32, CI 1.00-1.76). Most children consumed breakfast (73%), lunch (58%) and dinner (82%) sitting at a table or bench and this was positively associated with higher SEP for all meal types (OR 1.61-2.37, p<0.05). Increased television (TV) viewing during meals was inversely associated with SEP (OR 0.63, CI 0.54-0.72). Less than half of children (36%) watched TV during meals more than once a day.

**Conclusions:** Australian families engage in many healthy mealtime behaviours. Evidence that parents share meals with children and place high value on mealtimes with children provides important opportunities for promoting healthy behaviours in families. The choice of eating location and the practice of viewing TV during mealtimes are examples of two such opportunities. Socioeconomic patterning of the location of mealtimes and TV viewing during meals may contribute to socioeconomic differences in dietary intakes and may be important targets for future health promotion.

**Additional file 1**

File format: Word document .docx

Title of data: Supplementary Table 1 *Family Meals with Young Kids survey questions, response scales and sources used to inform development of questions and response items*

Description of data: Summary of survey question, corresponding response scales and sources used to inform the development of each question analyzed in this study.

**Keywords**

Young children

Family meal

Mealtime characteristics

Family food environment

Socioeconomic

Australia

## Background

Research into children’s eating behaviours is a priority given increasing evidence that health in adult life is influenced by dietary habits and behaviours commencing in childhood [[1](#_ENREF_1), [2](#_ENREF_2)]. Dietary behaviours develop in the early years of life and evidence suggests these track across most life stages [[3](#_ENREF_3)]. The health impacts of current trends in children’s diet and physical activity behaviours are evident with one quarter of Australian children aged two to 17 years overweight or obese [[4](#_ENREF_4)]. Many Australian children are not eating the recommended number of serves of fruit and vegetables for optimal health [[5](#_ENREF_5)] and more than one third of their daily energy intake is derived from discretionary foods [[4](#_ENREF_4)]. Given the association between diet, body weight and health, establishing healthy habits during the first years of life is crucial and underscores why improving the diets of young children should be public health priority.

Young children (in this paper defined as those six months to six years of age) share their food environment with caregivers (namely parents) and siblings [[6](#_ENREF_6)]. This shared ‘family food environment’ is perhaps the most important influence on children’s dietary intakes [[7](#_ENREF_7)] and therefore, provides an important target setting for improving diets and eating behaviours among young Australian children.

The family food environment is where food behaviours are initially developed and reinforced [[8](#_ENREF_8), [9](#_ENREF_9)]. It incorporates a cluster of potential parental influences on children’s diets, which offer opportunity for influencing dietary intakes among young children, particularly during shared family mealtimes (breakfast, lunch, dinner and snacks). Research in older children has indicated that many characteristics of family meals, such as frequency [[10](#_ENREF_10)], setting [[11](#_ENREF_11)] and the importance parents place on family meals [[12](#_ENREF_12)] are important however, these characteristics have not been examined in younger Australian children. Most research in this area has focused on the frequency of family meals in older children as a correlate of children’s psychological wellbeing or nutrient intakes. This body of research broadly suggests that both domains are positively associated with increased family meal frequency [[10](#_ENREF_10), [13](#_ENREF_13), [14](#_ENREF_14)].

Less research has been conducted regarding younger children with a smaller body of evidence suggesting that increased frequency of family meals is associated with higher intakes of fruit and vegetables [[6](#_ENREF_6), [15-17](#_ENREF_15)]. The only Australian study to have measured the frequency of family meals in children younger than six years reported that approximately 60% of families ate together every night [[16](#_ENREF_16)]. No studies within this age group in Australia or internationally have assessed family meals at times other than the evening meal. Given that young children tend to eat many small meals throughout the day, assessing family meal frequency across the day is important to inform where nutrition promotion efforts within the family food environment will be best targeted.

Information regarding other characteristics of Australian family mealtime practices is also needed. For example, eating location is considered to be an important characteristic of family meals, with eating while sitting at a table reported to be associated with younger children’s increased fruit and vegetable consumption [[11](#_ENREF_11)], appropriate portion sizes [[18](#_ENREF_18)], social engagement between parents and children [[19](#_ENREF_19)], and reduced access to TV viewing during meals [[20](#_ENREF_20)]. Conversely, eating in locations not specifically for dining has been associated with poorer diet quality [[20](#_ENREF_20)], and eating the family meal while watching television (TV) is consistently reported to be associated with poorer dietary intakes in this group [[15](#_ENREF_15), [21](#_ENREF_21)]. Australian data suggests that over a third of Australian children, aged four to twelve years, have the TV on during the evening meal [[22](#_ENREF_22)] however, information about eating location focussing on children under six years of age has not previously been reported either in Australia or internationally.

Given that health outcomes are known to be socioeconomically patterned [[23](#_ENREF_23)], it is important to assess family mealtime behaviours across socioeconomic circumstance as this may assist in targeting health promotion initiatives. Amongst older children, low socioeconomic position (SEP) has been shown to be associated with poorer nutrient intakes [[24](#_ENREF_24), [25](#_ENREF_25)], higher Body Mass Index (BMI) [[26](#_ENREF_26)], and decreased accessibility, purchasing and consumption of healthy foods [[24](#_ENREF_24), [27](#_ENREF_27)]. Television viewing during mealtimes appears to be inversely associated with SEP [[28](#_ENREF_28)] while other mealtime practices, such as purchasing takeaway foods for the evening meal [[28](#_ENREF_28)], reduced availability of supplies for meal preparation [[29](#_ENREF_29)] and eating in rooms not specifically designed for dining [[20](#_ENREF_20)], have also been associated with lower SEP. Evidence regarding associations between characteristics of family mealtimes with young children and SEP is mixed [[28](#_ENREF_28), [13](#_ENREF_13), [30](#_ENREF_30)]. Furthermore, the socioeconomic patterning of family mealtime behaviours, such as the proportion of children eating meals with their family over the course of the day, common locations in which children eat their meals, parents’ perceived importance of family meals, and whether parents and children are eating the same food during family meals, has not previously been assessed in any age group in Australia, or in this age group internationally.

The aim of this study was to describe the characteristics of mealtime behaviours among Australian families with children aged six months to six years, and to assess whether these mealtime behaviours were associated with SEP.

## Methods

*The Family Meals with Young Kids study* was conducted online, with recruitment via Australian Facebook sites and parent related blogs, the owners of which were invited to voluntarily advertise a short description of the study and the survey web link on their websites or Facebook pages. Active advertisement (contacting potential advertisers and having them post a link) and participant follow up ran for 7 weeks and required minimal researcher time. Participants were eligible to participate if they were the parent of a pre-school child aged between six months and to six years, living in Australia and with sufficient English language skills to complete the survey. Participants were asked to answer survey questions about their youngest child within this age range only. Eligibility checks were included in the online consent form. Participants were required to consent before answering survey questions. The survey platform SurveyMonkey® was utilized.

To maximize participation and completion of the survey, the majority of survey questions were not compulsory. Therefore, response numbers to each question vary. Ethics was approved through Deakin University HEAG-H 55_2014.

**Survey measures**

Mealtime characteristics measured included; location in which breakfast, lunch, dinner and snacks are eaten; TV viewing during meals; time and duration of family meals; parental modelling of food consumption during the evening meal; overall parent rated importance of family meals; and frequency of shared meals. Given the lack of consistency in survey measures assessing this topic area, the most appropriate measure of frequency of family meals has not been determined. Therefore, family meal frequency was measured using two separate, previously reported definitions; ‘how often do you and [your child] eat [breakfast/lunch/dinner/snacks] together’ (adapted from child surveys [[14](#_ENREF_14), [31](#_ENREF_31)]) and ‘how often does everyone who lives in the house eat [breakfast/lunch/dinner/snacks] together’ [[13](#_ENREF_13), [32](#_ENREF_32)]. A summary of the survey questions and response items is displayed in Supplementary Table 1 (Additional File 1). In addition to these items, the weekly frequency of family meals was assessed by summing breakfast, lunch, dinner and one snack, each day, over seven days. These were then summed and a total frequency from 28 possible eating occasions across the week (assessed for both ‘eat(ing) these meals with your child’ and ‘everyone who lives in your house eat(ing) these meals together’). Given that ‘snacks’ were measured as a group of eating occasions across the day throughout the survey and then condensed to one occasion per day for this analysis, results represent a modest view of frequency. The weekly frequency of watching TV viewing during meals was also assessed using this method.

The education of the responding parent was used in this study as a proxy for SEP. Maternal education has been shown to be a valid and reliable indicator of SEP [[33](#_ENREF_33)] and given that most participants were mothers, many employed part time or not working (making income less appropriate), education was considered to be the most valid proxy of SEP for this study. Maternal education is also known to be an important predictor of child diet [[34](#_ENREF_34)]. For the purpose of analyses, the responding parents’ education level, was dichotomised to university educated or non-university educated.

**Reliability**

A number of papers assessing family meals were used to inform the development of survey questions and response options [[18-20](#_ENREF_18), [28](#_ENREF_28), [31](#_ENREF_31), [35-41](#_ENREF_35)]. Given that a number of items were purpose designed or not previously used in this age group, a test-retest study was also conducted to measure the reliability of survey questions. This included a subsample of 54 study participants who completed a repeat survey one to two weeks after their initial survey completion.

**Statistical Analysis**

Data analysis was conducted using STATA® 12.0. Associations with parental education were assessed using linear regression (continuous variables), binary logistic regression (dichotomous variables), and ordered logistic regression (ordered categorical data). Weekly frequency of TV viewing during meals was analyzed using Poisson regression, given the skewed data distribution. All analyses were adjusted for child age, as this variable was considered likely to impact outcomes assessed.

## For the purposes of analyzing location of family meals by parental education, data was dichotomized to compare optimal family meal location (sitting at table/bench) with less than optimal locations (‘sitting on couch/floor’, ‘moving around the house’, ‘sitting at high chair (not at table/bench)’ and ‘in car’). This categorisation was informed by literature suggesting that eating at the table promotes healthier nutrition and psychosocial related outcomes, in comparison with other locations. Some locationsdeemed as neither optimal, nor less than optimal (‘’, ‘at childcare’, ‘at home of friend/ family member’, ‘outside’ and ‘other’), were excluded from the analysis. Children under one year were also excluded from the analysis of eating locations because it is likely that very young children’s eating locations would be influenced by motor skills and postural control.

## Results

**Participant demographics**
Participant demographics can be found in Table 1. Nine hundred and ninety two participants gave informed consent to participate in this online study and completed at least one of the survey items relevant to this analysis.

**Time and duration of the evening meal (not reported in tables)**
The most commonly reported times for the evening meal (n=737 respondents) were 6pm (28%), 5.30pm (26%) and 6.30pm (17%). The remaining 29% of families ate dinner between 4.30pm and 9pm. Eating dinner later in the evening was not associated with parental education level (β-coefficient -0.04, CI -0.14-0.05, p 0.37). Reliability was considered to be good (ICC 0.84) [[42](#_ENREF_42)]. Time taken to eat the evening meal ranged from 10 to 60 minutes (n=864). Half of all evening meals were reported to last on average 30 minutes. When assessing duration of family meals (n=792), longer duration was not associated with parental education (OR 0.82, CI 0.61-1.09, p 0.16). Reliability was considered to be moderate (ICC 0.74) [[42](#_ENREF_42)].

**Parent eating the same food as their child during the evening meal and parent perceived importance of family meals (not reported in tables)**Around seven in ten parents reported eating the same food as their child on at least five nights per week. Frequency of eating the same food was not associated with parental education (OR 0.97, CI 0.74-1.27, p 0.68). The ICC was considered to be good (ICC 0.77) [[42](#_ENREF_42)].

Most parents reported that family meals were ‘quite important’ (34%) or ‘very important’ (58%). Participants with higher education level rated family meals as more important although this was not significant (OR 1.32, CI 0.99-1.75, p 0.057). Reliability was considered to be moderate (ICC 0.68) [[42](#_ENREF_42)].

**Mealtime frequency**
Mealtime frequency data is presented in Table 2. The frequency of family meals per week varied by meal types (n=958). The most frequently reported family meal was dinner, with 77% of children sharing this meal with at least one parent, on at least five evenings per week and 6% on less than one evening per week. Most parents (59%) reported eating dinner with their child every evening. The least frequently reported meal type shared between parent and child was snacks, with 61% of children eating snacks with their parent fewer than five days per week. Higher parental education was not significantly associated with family meal frequency for any of the meal types when family meals were defined as a meal shared by the respondent and their child. However, when family meals were defined as ‘everyone who lives in the house eating together’, higher parental education was associated with a lower frequency of family dinners (OR 0.70, CI 0.54-0.92, p 0.01). When the frequency of a child eating a meal with the respondent was summed across the week, 4% of children were found to be eating meals with their parent on fewer than seven occasions per week (i.e. average < once per day) and 43% of children were eating meals with their parent on 21-28 occasions per week (i.e. average ≥ three times per day). There were no differences in summed family meal frequency by SEP (β-coefficient -0.22, CI -1.20-0.76, p 0.66). Reliability was considered moderate (ICC 0.72) for household and good for parent and child (ICC 0.85) [[42](#_ENREF_42)].

**Mealtime locations**
Table 3 compares the proportion of children who most frequently ate breakfast, lunch, dinner and snacks in the locations deemed optimal and less than optimal. Higher parental education was significantly associated with the likelihood of eating at a table or bench, (compared to other locations) for all meals except dinner. Reliability was considered to range between moderate to substantial (Kappa 0.56-0.77) [[42](#_ENREF_42)].

**Television viewing during mealtimes**

Higher parental education was significantly and inversely associated with less frequent TV viewing during breakfast, lunch, dinner and snacks (OR 0.38-0.60, p<0.00).

Table 3 outlines the proportion of children watching TV during meals, for each meal and the weekly frequency. On average, sixty four percent of children watched TV during one or fewer mealtimes per day. The likelihood of this decreased with higher parental education (IRR 0.63, CI 0.54-0.72, p<0.00). Intraclass correlations were considered to be good (ICC 0.75-0.96) [[42](#_ENREF_42)].

**Reliability**

Reliability of survey items was considered moderate to good/substantial [[42](#_ENREF_42)]. Intraclass correlations (ICCs) for all ordered categorical variables, and Kappa for non-ordered categorical variables have been presented throughout the relevant results sections of this paper.

## Discussion

This study has described mealtime behaviours of young Australian children, aged six months to six years, by assessing some of the less commonly identified characteristics of family meals, likely to be associated with improved diet.

Given that the evening meal is the most commonly researched family meal type [[21](#_ENREF_21), [37](#_ENREF_37), [43](#_ENREF_43)], it is important to understand its frequency, particularly as in Australia, this is the meal families traditionally share. Results from the current study reflect this, with shared evening meals the most commonly reported meal, and the meal most parents share with their child every night. This finding is consistent with the only other Australian study reporting family meal frequency within this age group [[16](#_ENREF_16)]. These two studies highlight that a large proportion of Australian families are frequently engaging in evening family meals. Importantly however, the current study also highlights that almost a quarter of parents reported eating with their child less than five evenings per week. This may be due to factors such as parental working hours, family size, varying ages and extra-curricular commitments of other siblings and or parents. Further research is warranted to determine what prevents families from eating meals together. Although the evidence base is smaller in younger children, regularly eating the evening meal together appears to be important for child health [[15](#_ENREF_15), [6](#_ENREF_6), [16](#_ENREF_16)]. Giving children every opportunity to be exposed to modelling at mealtimes, particularly the modelling of the consumption of vegetables may be an important way to develop and encourage healthy eating behaviours and habits. To our knowledge, this is the first study within this age group to assess family meal frequency in Australia, by differences in Socioeconomic patterning. Fewer family meals with everyone from the household present were found to be associated with the responding parent having a university education. Higher SEP is not often associated with less healthy behaviours however, this finding is consistent with some of the previous research focused on SEP and family meal frequency [[44](#_ENREF_44), [45](#_ENREF_45), [13](#_ENREF_13)]. An interplay of factors such as such as parental working hours, family size and partners’ education level is likely to influence family meal frequency and the association with SEP, but further research is warranted to better understand this.

Apart from the evening meal, it is also important to understand the frequency and location of family meals at other times of the day [[41](#_ENREF_41)] as these may also offer opportunities for children to establish healthy eating behaviours. Snacks as a shared mealtime may provide the best potential for nutrition promotion given that, as the current study identified, snacks are least frequently shared with parents and most commonly consumed in a less than optimal location. Changing children’s snacking behaviours, namely by increasing fruit and vegetable intakes to displace discretionary foods, is important in the context of improving child health. Research from the US suggests that changes in snacking behaviour may be a contributing factor to increasing weight trends in young children [[46-48](#_ENREF_46)] and should be an area for targeted health promotion. Although it may not be convenient for parents to eat snacks together as a family, especially for working parents, more benefits may be conferred to children if parents viewed snack time as an important time for eating together, role modelling, and opportunity for the consumption of nutritious foods.

The location of eating is also an important part of mealtime context for children [[41](#_ENREF_41)]. Previous studies have briefly explored family traditional dining locations, such as sitting at the table, kitchen or dining area [[20](#_ENREF_20), [40](#_ENREF_40), [16](#_ENREF_16)] yet very few studies have assessed the most common locations that children consume different meal types throughout the day. In Australia, location of other shared meals has not been assessed for this age group, until now. In the current study, children frequently ate in the car or while moving around the house. This was particularly evident for snack consumption. This indicates that the benefits of both sharing the mealtime and eating in an ‘optimal’ location are not being conferred, offering a two-fold disadvantage. Thus, promotion of nutrition through mealtime settings should focus on location as an important aspect of family mealtime occasions. Furthermore, the current study indicated that parents of higher SEP were more likely to report that their child ate at a table or bench for each meal type, consistent with the one other related study [[49](#_ENREF_49)]. Socioeconomic differences are likely to be multifaceted in nature and may be linked to the socioeconomic patterning of parent rated importance of family meals, as well as practical considerations such as having a room in the house allocated to dining [[20](#_ENREF_20)].

In addition to location, the context of eating is an important part of mealtimes for children [[41](#_ENREF_41)]. The frequency of children watching TV whilst eating meals is important to understand, given this has been associated with consuming more discretionary foods and fewer fruits and vegetables [[15](#_ENREF_15), [50](#_ENREF_50)]. In the current study around one third of parents reported that their child watched TV during meals at least once per day. Similarly, in an Australian sample of four to 12 year old children, 41% of families had the TV on during every evening meal [[22](#_ENREF_22)]. Another Australian study reported that three to five year old children ate dinner in front of the TV an average of 2.2 evenings per week [[16](#_ENREF_16)]. It is important to acknowledge that families of less educated parents in the current study were more likely to watch TV during meals, highlighting the importance of tailoring family meals interventions to groups most at risk of poor diet and related behaviours. Exploring the reasons behind the socioeconomic patterning of mealtime behaviours, in particular TV viewing during meals and eating location will be an important strategy for appropriately targeting lower SEP families in promoting healthy family mealtimes.

The participant sample included more highly educated participants (71% tertiary educated) than the average Australian adult population [[51](#_ENREF_51)]. Although this is a common occurrence in research [[52](#_ENREF_52)], it means results may not be generalizable to the whole population. Recruiting online was an efficient and effective method however, more research into ways to use this recruitment method to reach more diverse groups would be valuable and would improve the generalisability of such research in future. A further recruitment challenge was that, the vast majority (97%) of participants were mothers, despite the fact that all parents were invited to participate in the study. This highlights the difficulty of recruiting fathers in research, and the importance of future research exploring the roles of fathers during family mealtimes [[53](#_ENREF_53)]. It is also acknowledged that online, self-report surveys can be affected by respondent interpretation of questions, social desirability bias and self-selection (non-random) bias. While efforts were made in study advertising to recruit participants with diverse views about family meals, the study may have attracted those with highest interest and motivation related to this topic. This study also required participants to have access to the internet. Though internet access in Australia is high (96% of families with children under 15 years in 2012-13 [[54](#_ENREF_54)]), those without internet access are unrepresented, and are most likely the lowest socioeconomic groups. Finally, this study is cross sectional and encompasses a large age range of children (pre-schoolers who have started solids). Research in this area would be strengthened by the use of longitudinal designs and studies that focus investigations within smaller age ranges.

An important strength of this study was the development of reliable, purpose designed questions. The test-retest analysis of these questions showed moderate to good/substantial reliability [[42](#_ENREF_42)] and as such provide reliable survey measures. Further, the online recruitment and survey design of this study enabled rapid, low cost data collection with all advertisement voluntarily (no cost) displayed on popular parenting online sites. Recruitment occurred over a period of less than three months.

## Conclusions

This study has added further insights into the understanding of family meals in the Australian context by exploring family meal frequency, common locations and TV viewing during mealtimes and their relationships with SEP, for children less than six years of age. Family meals appear to be an important and frequent occurrence within Australian families. This highlights the relevance and potential for promoting healthy behaviours targeting the family meal setting. Particularly, as there are few studies internationally which have specifically focussed on using the family meal setting as an opportunity for nutrition promotion interventions to improve child diets [[55](#_ENREF_55)]. Understanding the diverse characteristics of family meals in Australia provides rationale for our selection of targets which aim to improve early childhood nutrition through mealtimes. The data presented in this paper suggest that a focus on mealtime location and TV viewing during meals, particularly in lower SEP families, is merited and will be useful to inform future nutrition promotion initiatives in Australia.

**List of abbreviations used**

Socioeconomic Position (SEP)

Television (TV)

Body Mass Index (BMI)

Odds Ratio (OR)

Confidence Interval (CI)

Intraclass Correlations (ICC)

Incident Rate Ratios (IRR)

## Declarations

**Ethics approval and consent to participate**

Ethics was approved through Deakin University HEAG-H 55_2014. To participate in this study, participants were required to read the online Plain Language Statement and give informed consent before progressing to the survey questions.

**Consent for publication**

Not applicable

**Availability of data and material**

The datasets generated and/or analysed during the current study are not publicly available because the study is not concluded, but may be available from the corresponding author on reasonable request.

**Competing interests**

The authors declare that they have no competing interests.

**Funding**

Funding was provided by Institute for Physical Activity and Nutrition, Deakin University.

**Authors’ contributions**

EL collected survey data, drafted the majority of the manuscript, performed data analysis and contributed to interpretation of data. AS lead study design, contributed to data analysis and interpretation of data and helped to draft the manuscript. KC contributed to study design, interpretation of data and helped to draft the manuscript. All authors read and approved the final manuscript.

**Acknowledgements**

The authors acknowledge the valuable contribution to this study made by Jennifer McCann, and also thank the study participants.

## References

1. Bellisle F. Why should we study human food intake behaviour? Nutrition, Metabolism and Cardiovascular Diseases. 2003;13(4):189-93.

2. Pearce J, Langley-Evans SC. The types of food introduced during complementary feeding and risk of childhood obesity: a systematic review. Int J Obes (Lond). 2013;37(4):477-85. doi:10.1038/ijo.2013.8.

3. Skinner JD, Carruth BR, Bounds W, Ziegler PJ. Children's food preferences: a longitudinal analysis. J Am Diet Assoc. 2002;102(11):1638-47.

4. Australian Bureau of Statistics. Australian Health Survey: Nutrition First Results - Foods and Nutrients, 2011-12. Report no: 4364.0.55.007. 2014.

5. Australian Bureau of Statistics. Australian Health Survey: Updated Results, 2011-2012. Report no: 4364.0.55.003. 2013.

6. Cooke LJ, Wardle J, Gibson EL, Sapochnik M, Sheiham A, Lawson M. Demographic, familial and trait predictors of fruit and vegetable consumption by pre-school children. Public Health Nutr. 2004;7(2):295-302.

7. Pearson N, Biddle SJ, Gorely T. Family correlates of fruit and vegetable consumption in children and adolescents: a systematic review. Public Health Nutr. 2009;12(02):267-83.

8. Arcan C, Neumark-Sztainer D, Hannan P, van den Berg P, Story M, Larson N. Parental eating behaviours, home food environment and adolescent intakes of fruits, vegetables and dairy foods: longitudinal findings from Project EAT. Public Health Nutr. 2007;10(11):1257-65.

9. Campbell K, Crawford D. Family food environments as determinants of preschool-aged children's eating behaviours: implications for obesity prevention policy. A review. Aust J Nutr Diet. 2001;58(1):19-25.

10. Gillman MW, Rifas-Shiman SL, Frazier AL, Rockett HR, Camargo Jr CA, Field AE et al. Family dinner and diet quality among older children and adolescents. Arch Fam Med. 2000;9(3):235.

11. Christian MS, Evans CE, Hancock N, Nykjaer C, Cade JE. Family meals can help children reach their 5 a day: a cross-sectional survey of children's dietary intake from London primary schools. J Epidemiol Community Health. 2013;67(4):332-8. doi:10.1136/jech-2012-201604.

12. McIntosh WA, Kubena KS, Tolle G, Dean WR, Jan JS, Anding J. Mothers and meals. The effects of mothers' meal planning and shopping motivations on children's participation in family meals. Appetite. 2010;55(3):623-8. doi:10.1016/j.appet.2010.09.016.

13. Neumark-Sztainer D, Hannan PJ, Story M, Croll J, Perry C. Family meal patterns: associations with sociodemographic characteristics and improved dietary intake among adolescents. J Am Diet Assoc. 2003;103(3):317-22.

14. Videon TM, Manning CK. Influences on adolescent eating patterns: the importance of family meals. J Adolesc Health. 2003;32(5):365-73. doi:10.1016/s1054-139x(02)00711-5.

15. Fitzpatrick E, Edmunds LS, Dennison BA. Positive effects of family dinner are undone by television viewing. Journal of the American Dietetic Association. 2007;107(4):666-71. doi:10.1016/j.jada.2007.01.014.

16. Wyse R, Campbell E, Nathan N, Wolfenden L. Associations between characteristics of the home food environment and fruit and vegetable intake in preschool children: a cross-sectional study. BMC public health. 2011;11:938-. doi:10.1186/1471-2458-11-938.

17. Hammons AJ, Fiese BH. Is frequency of shared family meals related to the nutritional health of children and adolescents? Pediatrics. 2011;127(6):e1565-74. doi:10.1542/peds.2010-1440.

18. Schwartz S, Benuck I. Strategies and suggestions for a healthy toddler diet. Pediatr Ann. 2013;42(9):181-3. doi:10.3928/00904481-20130823-09.

19. Wansink B, van Kleef E. Dinner rituals that correlate with child and adult BMI. Obesity (Silver Spring). 2014;22(5):E91-5. doi:10.1002/oby.20629.

20. Skafida V. The family meal panacea: exploring how different aspects of family meal occurrence, meal habits and meal enjoyment relate to young children's diets. Sociol Health Illn. 2013;35(6):906-23. doi:10.1111/1467-9566.12007.

21. Sweetman C, McGowan L, Croker H, Cooke L. Characteristics of family mealtimes affecting children's vegetable consumption and liking. J Am Diet Assoc. 2011;111(2):269-73. doi:10.1016/j.jada.2010.10.050.

22. van Zutphen M, Bell AC, Kremer PJ, Swinburn BA. Association between the family environment and television viewing in Australian children. J Paediatr Child Health. 2007;43(6):458-63.

23. Department of Health. Victorian Population Health Survey 2010. In: of SG, Victoria, editors. Victorian Department of Health, Melbourne, Victoria: Prevention and Population Health Branch 2012.

24. Zarnowiecki DM, Dollman J, Parletta N. Associations between predictors of children's dietary intake and socioeconomic position: a systematic review of the literature. Obes Rev. 2014;15(5):375-91. doi:10.1111/obr.12139.

25. Xie B, Gilliland FD, Li Y-F, Rockett HRH. Effects of Ethnicity, Family Income, and Education on Dietary Intake among Adolescents. Prev Med. 2003;36(1):30-40. doi:10.1006/pmed.2002.1131.

26. Cameron AJ, Ball K, Pearson N, Lioret S, Crawford DA, Campbell K et al. Socioeconomic variation in diet and activity-related behaviours of Australian children and adolescents aged 2-16 years. Pediatr Obes. 2012;7(4):329-42. doi:10.1111/j.2047-6310.2012.00060.x.

27. Inglis V, Ball K, Crawford D. Does modifying the household food budget predict changes in the healthfulness of purchasing choices among low- and high-income women? Appetite. 2009;52(2):273-9. doi:<http://dx.doi.org/10.1016/j.appet.2008.10.005>.

28. Campbell K, Crawford D, Jackson M, Cashel K, Worsley A, Gibbons K et al. Family food environments of 5− 6‐year‐old‐children: Does socioeconomic status make a difference? Asia Pac J Clin Nutr. 2002;11(s3):S553-S61.

29. Appelhans BM, Waring ME, Schneider KL, Pagoto SL. Food preparation supplies predict children's family meal and home-prepared dinner consumption in low-income households. Appetite. 2014;76:1-8.

30. Allen TD, Shockley KM, Poteat LF. Workplace factors associated with family dinner behaviors. Journal of Vocational Behavior. 2008;73(2):336-42. doi:10.1016/j.jvb.2008.07.004.

31. Woodruff SJ, Kirby AR. The associations among family meal frequency, food preparation frequency, self-efficacy for cooking, and food preparation techniques in children and adolescents. Journal of nutrition education and behavior. 2013;45(4):296-303. doi:10.1016/j.jneb.2012.11.006.

32. Koszewski W, Behrends D, Nichols M, Sehi N, Jones G. Patterns of family meals and food and nutrition intake in limited resource families. Family and Consumer Sciences Research Journal. 2011;39(4):431-41.

33. Winkleby MA, Jatulis DE, Frank E, Fortmann SP. Socioeconomic status and health: how education, income, and occupation contribute to risk factors for cardiovascular disease. Am J Public Health. 1992;82(6):816-20.

34. Zarnowiecki D, Ball K, Parletta N, Dollman J. Describing socioeconomic gradients in children's diets - does the socioeconomic indicator used matter? The international journal of behavioral nutrition and physical activity. 2014;11(1):44-. doi:10.1186/1479-5868-11-44.

35. Anderson SE, Must A, Curtin C, Bandini LG. Meals in Our Household: reliability and initial validation of a questionnaire to assess child mealtime behaviors and family mealtime environments. J Acad Nutr Diet. 2012;112(2):276-84.

36. Centre for Physical Activity and Nutrition (C-PAN). About The InFANT Extend Program. <http://www.deakin.edu.au/health/cpan/infant-extend/index.php>. Accessed 19th September 2014.

37. Neumark-Sztainer D, Wall M, Fulkerson JA, Larson N. Changes in the frequency of family meals from 1999 to 2010 in the homes of adolescents: trends by sociodemographic characteristics. The Journal of adolescent health : official publication of the Society for Adolescent Medicine. 2013;52(2):201-6. doi:10.1016/j.jadohealth.2012.06.004.

38. Haines J, McDonald J, O'Brien A, Sherry B, Bottino CJ, Schmidt ME et al. Healthy Habits, Happy Homes: randomized trial to improve household routines for obesity prevention among preschool-aged children. JAMA Pediatrics. 2013;167(11):1072-9. doi:10.1001/jamapediatrics.2013.2356.

39. Mamun AA, Lawlor DA, O'Callaghan MJ, Williams GM, Najman JM. Positive maternal attitude to the family eating together decreases the risk of adolescent overweight. Obes Res. 2005;13(8):1422-30.

40. Gallegos D, Dziurawiec S, Fozdar F, Abernethie L. Adolescent experiences of ‘family meals’ in Australia. Journal of Sociology. 2011;47(3):243-60.

41. Mak TN, Prynne CJ, Cole D, Fitt E, Roberts C, Bates B et al. Assessing eating context and fruit and vegetable consumption in children: new methods using food diaries in the UK National Diet and Nutrition Survey Rolling Programme. The international journal of behavioral nutrition and physical activity. 2012;9:126. doi:10.1186/1479-5868-9-126.

42. Sim J, Wright C. Research in Health Care: Concepts, Designs and Methods. United Kingdom: Nelson Thornes Ltd 2000.

43. Eisenberg ME, Neumark-Sztainer D, Fulkerson JA, Story M. Family meals and substance use: is there a long-term protective association? The Journal of adolescent health : official publication of the Society for Adolescent Medicine. 2008;43(2):151-6. doi:10.1016/j.jadohealth.2008.01.019.

44. Moser A, Chen SE, Jilcott SB, Nayga RM. Associations between maternal employment and time spent in nutrition-related behaviours among German children and mothers. Public Health Nutr. 2012;15(7):1256-61. doi:10.1017/S1368980011003375.

45. Bauer KW, Hearst MO, Escoto K, Berge JM, Neumark-Sztainer D. Parental employment and work-family stress: associations with family food environments. Social science & medicine. 2012;75(3):496-504. doi:10.1016/j.socscimed.2012.03.026.

46. Ford CN, Slining MM, Popkin BM. Trends in Dietary Intake among US 2-to 6-Year-Old Children, 1989-2008. J Acad Nutr Diet. 2013;113(1):35-42. doi:10.1016/j.jand.2012.08.022.

47. Piernas C, Popkin BM. Trends In Snacking Among US Children. Health Aff (Millwood). 2010;29(3):398-404. doi:10.1377/hlthaff.2009.0666.

48. Australian Bureau of Statistics. Australian Health Survey: Nutrition First Results - Foods and Nutrients, 2011-12, cat no. 4364.0.55.007. Canberra2014.

49. Attorp A, Scott JE, Yew AC, Rhodes RE, Barr SI, Naylor P-J. Associations between socioeconomic, parental and home environment factors and fruit and vegetable consumption of children in grades five and six in British Columbia, Canada. BMC public health. 2014;14:150-. doi:10.1186/1471-2458-14-150.

50. Coon KA, Goldberg J, Rogers BL, Tucker KL. Relationships between use of television during meals and children's food consumption patterns. Pediatrics. 2001;107(1). doi:10.1542/peds.107.1.e7.

51. Australian Bureau of Statistics. Education and Work, Australia. Report no: 6227.0. Canberra2013.

52. Batterham PJ. Recruitment of mental health survey participants using Internet advertising: content, characteristics and cost effectiveness. Int J Methods Psychiatr Res. 2014(2):184. doi:10.1002/mpr.1421/abstract.

53. Walsh AD, Cameron AJ, Hesketh KD, Crawford D, Campbell KJ. Associations between dietary intakes of first-time fathers and their 20-month-old children are moderated by fathers' BMI, education and age. Cambridge University Press; 2015.

54. Australian Bureau of Statistics. Household Use of Information Technology, Australia, 2012-13. Report no: 8146.0. Canberra2014.

55. Flattum C, Draxten M, Horning M, Fulkerson JA, Neumark-Sztainer D, Garwick A et al. HOME Plus: Program design and implementation of a family-focused, community-based intervention to promote the frequency and healthfulness of family meals, reduce children's sedentary behavior, and prevent obesity. International Journal of Behavioral Nutrition & Physical Activity. 2015;12:1.

**Table 1** *Demographic characteristics of participants*

| **Parent characteristic (number of responses to item)** | **n** | **Percent** |
| --- | --- | --- |
| **Age (n =877)** Mean age 35 years (range 19-59 years) |  | |
| **More than one child in the household (n=879)** | 629 | 72% |
| **Relationship to child (n = 992)** |  |  |
| Mother | 963 | 97% |
| Father | 25 | 3% |
| Other | 4 | 0.40% |
| **Country of birth (n = 902)** |  |  |
| Australia | 748 | 83% |
| Other | 154 | 17% |
| **Current Marital Status (n = 910)** |  |  |
| Married | 741 | 81% |
| Defacto | 130 | 14% |
| Separated | 19 | 2% |
| Divorced/widowed/never married | 20 | 2% |
| **Highest level of completed schooling (n = 910)** |  |  |
| ≤ Year 12 or equivalent | 96 | 10% |
| Trade / apprenticeship (e.g. hairdresser, chef) | 7 | 0.80% |
| Certificate / diploma (e.g. childcare, technician) | 160 | 18% |
| University degree | 364 | 40% |
| Higher University degree (e.g. Graduate Diploma, Masters) | 283 | 31% |
| **Current main daily activities (n = 910)** |  |  |
| On maternity/ paternity leave | 135 | 15% |
| Working full-time | 112 | 12% |
| Working part-time | 380 | 42% |
| Studying full-time | 16 | 2% |
| Home duties full time | 235 | 26% |
| Other | 32 | 3% |
| **Child characteristics (number of responses to item)** | **n** | **Percent** |
| **Age (n = 992)** Mean age 2.5 years (range 0.5-5.9 years) |  | |
| **Gender** |  |  |
| Male | 521 | 53% |
| Female | 471 | 47% |

**Table 2** *Frequency of family meals with ‘respondent and child’ or ‘everyone who lives in the house’ eating together and comparison by SEP (responding parent education level)*

|  | **Breakfast** | **Lunch** | **Dinner** | **Snacks** |
| --- | --- | --- | --- | --- |
| **Respondent and child eating together** | |  |  |  |
| < 1 day/week | 8% | 5% | 6% | 8% |
| 1-2 days/week | 12% | 19% | 7% | 19% |
| 3-4 days/week | 17% | 34% | 10% | 35% |
| 5-6 days/week | 21% | 23% | 18% | 18% |
| 7 days/week | 43% | 20% | 59% | 20% |
| **Odds ratios (CI)** | 1.05 (0.79-1.39) | 0.83 (0.62-1.20) | 0.77 (0.57-1.03) | 1.02 (0.78-1.34) |
| **Everyone who lives in the house eating together** | | |  |  |
| < 1 day/week | 17% | 14% | 6% | 17% |
| 1-2 days/week | 37% | 63% | 12% | 52% |
| 3-4 days/week | 17% | 15% | 14% | 21% |
| 5-6 days/week | 11% | 5% | 24% | 6% |
| 7 days/week | 18% | 3% | 43% | 4% |
| **Odds ratios (CI)** | 1.20 (0.93-1.56) | 1.20 (0.74-1.39) | 0.70 (0.54-0.92)* | 0.91 (0.69-1.20) |
| **Sum of frequencies across the week  (occasions per week)** | **Respondent and child** |  | **Everyone who lives in the house** | |
| <7 | 4% |  | 17% |  |
| 7-<14 | 16% |  | 48% |  |
| 14-<21 | 37% |  | 26% |  |
| 21-28 | 43% |  | 9% |  |
| **Coefficient (CI)** | -0.22 (1.20-0.76) | | -0.29 (CI -1.13-0.55) | |
| *Indicates p≤0.05 | | | | |

**Table 3** *Percentage of children watching TV during meals, for each meal and the summed weekly frequency (n=946), and the proportion of children eating meals in a recommended location and non-recommended locations (n=943) and comparison by SEP (responding parent education level) (n=606).*

|  | All children | University educated parent | Non-university educated parent |
| --- | --- | --- | --- |
| **Breakfast** (OR 0.55, CI 0.41-0.72)* | | | |
| <1 day/week | 60% | 66% | 48% |
| 1-4 days/week | 24% | 20% | 32% |
| ≥5 days/week | 16% | 14% | 20% |
| **Lunch** (OR 0.38, CI 0.28-0.50)* | | | |
| <1 day/week | 66% | 73% | 50% |
| 1-4 days/week | 29% | 24% | 41% |
| ≥5 days/week | 5% | 7% | 9% |
| **Dinner** (OR 0.60, CI 0.45-0.80)* | | | |
| <1 day/week | 66% | 70% | 58% |
| 1-4 days/week | 24% | 22% | 28% |
| ≥5 days/week | 10% | 8% | 14% |
| **Snacks** (OR 0.50, CI 0.38-0.65)* | | | |
| <1 day/week | 38% | 42% | 28% |
| 1-4 days/week | 50% | 48% | 53% |
| ≥5 days/week | 12% | 10% | 19% |
| **Sum of frequencies across the week** (IRR 0.63, CI 0.54-0.72)* | | | |
| <7 occasions/week | 64% | 69% | 54% |
| 7-<14 occasions/week | 22% | 21% | 24% |
| 14-<21 occasions/week | 10% | 8% | 14% |
| 21-28 occasions/week | 3% | 2% | 7% |
|  | **Proportion of children eating in Recommended location**** | **Proportion of children eating in Non-recommended locations***** | **Comparison by SEP (OR (CI))** |
| **Breakfast** | 73% | 24% | 1.86 (1.27-2.71)* |
| **Lunch** | 58% | 21% | 2.35 (1.60-3.51)* |
| **Dinner** | 83% | 14% | 1.61 (0.99-2.61) |
| **Snacks** | 24% | 62% | 1.97 (1.33-2.91)* |
| *Indicates p≤0.05  **Sitting at table/bench at home ***Sitting on couch/ floor, In the car, Sitting at high chair (not at table/ bench), Moving around the house | | | |
